# Pesticide-specific case fatality and clinical outcomes among poisoning cases in Tanzania: a retrospective cross-sectional study

**DOI:** 10.3389/ftox.2026.1882231

**Published:** 2026-07-23

**Authors:** Raphael Mwezi, Joseph Bukalasa, Fredrick Otieno, Rosevera Kombo, Grivin Ochola, Florida Muro, Anicet Honest, Sarah Urasa, Mark Davis, Michael Eddleston, Marieke Dekker, William Howlett, John-Mary Vianney

**Affiliations:** 1 Tanzania Plant Health and Pesticide Authority (TPHPA), Directorate of Pesticide, Arusha, Tanzania; 2 Department of Global Health and Biomedical Sciences, Nelson Mandela African Institution of Science and Technology, Arusha, Tanzania; 3 Food and Agriculture Organization of the United Nations (FAO), UN Liaison Office, Dodoma, Tanzania; 4 Centre for Environment Justice and Development (CEJAD), Nairobi, Kenya; 5 Department of Internal Medicine, Kilimanjaro Christian Medical Centre, Moshi, Tanzania; 6 Department of Internal Medicine, KCMC University, Moshi, Tanzania; 7 Centre for Pesticide Suicide Prevention Queen’s Medical Research Institute, The University of Edinburgh, Edinburgh, United Kingdom

**Keywords:** case fatality, highly hazardous pesticides, mortality, paraquat, pesticide poisoning, poisoning outcomes, Tanzania, toxicovigilance

## Abstract

**Background:**

Pesticide poisoning remains a major public health challenge in low- and middle-income countries, particularly where highly hazardous pesticides (HHPs) remain accessible and toxicovigilance systems are limited. Although Tanzania has recently initiated regulatory actions targeting HHPs, evidence regarding pesticide-specific mortality patterns and clinical outcomes remains scarce. This study assessed clinical outcomes, pesticide-specific case fatality, and mortality risk among pesticide poisoning cases reported in selected Tanzanian hospitals.

**Methods:**

A retrospective cross-sectional study was conducted using hospital records from Regional Referral Hospitals and District Hospitals in Arusha, Kilimanjaro, Morogoro, Iringa, and Mwanza regions between January 2017 and November 2024. Cases were identified through hospital poisoning records, emergency department registers, admission records, and medical files. Information on demographic characteristics, exposure type, pesticide category, and clinical outcomes was extracted using structured electronic forms. Descriptive statistics summarize poisoning characteristics and outcomes. Associations between demographic factors and clinical outcomes were assessed using chi-square tests. Binary logistic regression was used to identify predictors of mortality, and adjusted odds ratios (aORs) with 95% confidence intervals (CIs) were reported.

**Results:**

A total of 635 pesticide poisoning cases were included in the analysis. Most patients were male (64.1%) and aged 16–35 years (61.3%). Intentional exposure accounted for 70.2% of cases, while unintentional exposure represented 20.3%. Most patients fully recovered (67.9%; 95% CI: 64.1–71.4), whereas 18.4% (95% CI: 15.5–21.6) died following pesticide poisoning. Paraquat poisoning demonstrated the highest case fatality (93.3%), followed by glyphosate (45.5%) and tefluthrin (33.3%). Compared with lower-risk pesticide exposures, paraquat poisoning was associated with markedly increased odds of death (aOR = 21.5; 95% CI: 9.4–48.2), while glyphosate poisoning was associated with elevated mortality risk (aOR = 5.2; 95% CI: 1.8–14.6). Pesticides with case fatality exceeding 10% contributed 87.3% of all recorded deaths.

**Conclusion:**

Mortality following pesticide poisoning in Tanzania was strongly concentrated among a small number of highly hazardous pesticides, particularly paraquat. These findings support continued restriction and removal of highly hazardous pesticides, strengthening of pesticide-specific surveillance systems, improved clinical toxicology capacity, and enhanced poisoning prevention strategies. Continued monitoring will be important to evaluate the public health impact of recent pesticide regulatory reforms in Tanzania.

## 1 Introduction

Pesticide poisoning remains a major global public health concern, particularly in low- and middle-income countries (LMICs) where agricultural production relies heavily on chemical pest control and regulatory oversight may be limited (World Health Organization, 2019). Globally, millions of pesticide poisoning cases occur annually, resulting in substantial morbidity and mortality among farming communities and rural populations (Gunnell et al., 2007). The burden is disproportionately concentrated in LMICs, where highly hazardous pesticides (HHPs) remain widely available, safe-use practices are often inadequate, and poison surveillance systems are underdeveloped (Food and Agriculture Organization of the United Nations FAO & World Health Organization WHO, 2014). Pesticides contribute substantially to both occupational poisoning and intentional self-poisoning. Previous studies have demonstrated that a relatively small number of highly hazardous pesticides account for a disproportionate share of poisoning-related deaths worldwide (Buckley et al., 2021; Mew et al., 2017). Studies reported that pesticide self-poisoning remains a major contributor to mortality in rural agricultural communities (Prasanth Kumar et al., 2024). Consequently, international organizations including the World Health Organization (WHO) and the Food and Agriculture Organization (FAO) have advocated for progressive restriction and replacement of highly hazardous pesticides as a key strategy for reducing pesticide-related mortality (Food and Agriculture Organization of the United Nations FAO & World Health Organization WHO, 2014).

Among highly hazardous pesticides, paraquat has consistently been identified as one of the most lethal compounds following acute poisoning (Eddleston, 2020; Prasanth Kumar et al., 2024; Sharma et al., 2015). Paraquat toxicity causes severe oxidative injury, particularly affecting the lungs, kidneys, liver, and cardiovascular system, frequently resulting in multi-organ failure and death despite medical treatment (Arsac et al., 2021; Commission, 2020; Langston, 2017). Organophosphate pesticides remain among the most commonly reported causes of pesticide poisoning globally because of their widespread agricultural use and their ability to induce cholinergic toxicity through inhibition of acetylcholinesterase activity (Chidiebere Uchendu, 2012; Cotton et al., 2018; Fuhrimann et al., 2022; Ramadori, 2023). Severe poisoning may lead to respiratory failure, seizures, cardiovascular complications, and death (Jokanović, 2018; Lee et al., 2012). Although glyphosate is generally considered less acutely toxic than paraquat, severe poisoning following intentional ingestion of concentrated formulations has also been associated with significant morbidity and mortality (Eddleston, 2020; Fuselier et al., 2023; Samsel and Seneff, 2016; Silva et al., 2019).

In Tanzania, agricultural intensification and expansion of commercial crop production have been accompanied by increasing pesticide use (Kapeleka et al., 2019; Lekei et al., 2014; Mwabulambo et al., 2018). Several studies have documented pesticide exposure and acute pesticide poisoning among farmers, agricultural workers, women, and adolescents in different parts of the country (Kapeleka et al., 2025; Lekei et al., 2020). However, most available studies have focused primarily on exposure patterns, poisoning incidence, or demographic characteristics rather than pesticide-specific mortality and clinical outcomes. Consequently, evidence regarding the relative lethality of individual pesticide categories and their contribution to poisoning-related mortality remains limited. Recognizing the public health risks associated with highly hazardous pesticides, Tanzania has recently implemented regulatory actions through the Tanzania Plant Health and Pesticides Authority (TPHPA). These measures include withdrawal of pesticide products containing active ingredients classified as highly hazardous pesticides and removal of long-expired pesticide registrations from the market. Such interventions are consistent with global recommendations promoting restriction of highly hazardous pesticides as an effective means of reducing pesticide-related mortality and suicide deaths (Gunnell et al., 2017; Lee et al., 2021). However, baseline evidence describing pesticide-specific mortality patterns remains essential for evaluating the effectiveness of these interventions and informing future regulatory decisions.

Furthermore, understanding the demographic distribution of intentional and unintentional pesticide poisoning is important for designing targeted prevention strategies (World Health Organization, 2021). Young adults frequently represent the most affected age group in many agricultural settings, particularly where pesticides are readily accessible for both occupational use and self-harm (Bonvoisin et al., 2020). Characterizing clinical outcomes and identifying pesticides associated with disproportionate mortality may therefore contribute to more effective public health interventions, clinical preparedness, and pesticide policy reforms. Therefore, this study aimed to assess clinical outcomes among pesticide poisoning cases reported in selected Tanzanian hospitals, determine pesticide-specific case fatality, evaluate pesticide-specific mortality risks, identify pesticides contributing disproportionately to poisoning-related deaths, and examine demographic and exposure-related factors associated with mortality.

## 2 Methodology

### 2.1 Study design and study setting

A retrospective cross-sectional study was conducted using pesticide poisoning records collected from Regional Referral Hospitals and District Hospitals located in five regions of Tanzania: Arusha, Kilimanjaro, Morogoro, Iringa, and Mwanza. These regions were purposively selected to provide geographical representation across major agricultural zones of the country and because they maintained accessible hospital records related to poisoning cases. The study reviewed pesticide poisoning cases reported between January 2017 and November 2024. Participating hospitals serve as referral facilities for large rural and urban populations and receive poisoning cases from surrounding districts and health facilities. The selected regions represent areas with substantial agricultural activities, including horticulture, cereal production, cash crop farming, and mixed farming systems where pesticide use is common.

### 2.2 Case identification and data collection

Cases were identified through a comprehensive review of hospital records, including emergency department registers, inpatient admission registers, poisoning surveillance registers, medical records departments, and discharge summaries. Hospital records containing diagnoses consistent with pesticide poisoning were screened using International Classification of Diseases, 10th Revision (ICD-10) poisoning codes, clinician diagnoses, and documented poisoning histories. Data were extracted using a structured electronic data collection form developed in Kobo Collect. Information collected included demographic characteristics (age and sex), region and health facility, type of pesticide involved, exposure circumstances, exposure type (intentional, unintentional, or unknown), and clinical outcomes at discharge. Where available, information regarding the specific pesticide agent involved was also recorded. The pesticide implicated in each poisoning episode was identified from available clinical documentation, including patient histories, caregiver reports, referral notes, clinician records, and pesticide containers presented during hospital admission when documented. Laboratory toxicological confirmation was not available because toxicology testing facilities were not routinely accessible in participating hospitals. Cases in which the specific pesticide could not be reliably identified were classified as “unknown or unspecified pesticide” and were retained in the analysis to minimize exclusion bias and provide a more comprehensive representation of pesticide poisoning cases encountered within the healthcare system.

### 2.3 Hospital selection, inclusion and exclusion criteria

Hospitals were selected based on the following criteria.

1. Availability of historical poisoning records.
2. Geographic representation of major agricultural regions.
3. Capacity to provide access to medical records for research purposes.
4. Approval from hospital administration and relevant authorities.

#### 2.3.1 Inclusion criteria

Cases were included if they.

- Were recorded between January 2017 and November 2024.
- Had a documented diagnosis of pesticide poisoning.
- Had sufficient information regarding exposure and clinical outcome.
- Were managed in participating hospitals.

#### 2.3.2 Exclusion criteria

Cases were excluded if they.

- Had unclear poisoning diagnoses.
- Involved non-pesticide toxic agents.
- Had missing outcome information.
- Contained duplicate records identified during data cleaning.

### 2.4 Data quality assurance

To ensure consistency and accuracy, data collection was conducted by trained research personnel using standardized data abstraction procedures. Prior to full-scale data collection, the electronic data collection tool (Kobo Collect) was piloted using a sample of hospital records to assess its functionality, completeness, and suitability for data extraction. Feedback from the pilot exercise was used to refine the tool and standardize data collection procedures across all participating sites. Several quality assurance measures were implemented throughout the data collection process. These included the use of standardized data abstraction guidelines, training of data collectors on study procedures and variable definitions, daily review of completed extraction forms, verification of questionable or inconsistent entries against original medical records, and routine consistency checks during data cleaning. Following data collection, all records were reviewed for completeness, logical consistency, and duplication prior to analysis. Records with incomplete information for specific variables were retained in the dataset whenever possible to maximize data utilization and were categorized as missing or unknown where appropriate.

### 2.5 Study variables

#### 2.5.1 Study variables

The exposure variables included sex (male or female), age group (0–15, 16–25, 26–35, 36–50, and ≥50 years), exposure type (intentional, unintentional, or unknown), and pesticide category. These variables were selected based on their epidemiological relevance and availability within the hospital records reviewed. The primary outcome variable was the clinical outcome at discharge, which was categorized as fully recovered, recovered with complications, death, transferred, absconded, or unknown outcome. For the purpose of logistic regression analysis, clinical outcomes were dichotomized into death and survival, with survival comprising all non-fatal outcomes.

Pesticide-specific case fatality (CF) was calculated as the proportion of deaths among all cases attributed to a given pesticide category using the formula:

$$\text{Case Fatality }\left(\%\right)=\left(\text{Number of Deaths}/\text{Total Number of Cases}\right)\times 100$$

To facilitate interpretation of toxicity patterns observed in the study population, pesticide categories were further grouped according to their observed case fatality rates. Pesticides with case fatality rates greater than 40% were classified as extreme risk, those with case fatality rates between 10% and 40% as high risk, those with case fatality rates between 5% and 10% as moderate risk, and those with case fatality rates below 5% as lower risk. These categories were developed solely for descriptive interpretation of the study findings and do not represent internationally recognized pesticide hazard classifications.

### 2.6 Statistical analysis

#### 2.6.1 Statistical analysis

Data were analyzed using R Statistical Software version 4.5.3 (R Foundation for Statistical Computing, Vienna, Austria). Descriptive statistics were used to summarize demographic characteristics, exposure circumstances, pesticide categories, and clinical outcomes. Categorical variables were summarized using frequencies, percentages, and corresponding 95% confidence intervals (95% CIs). Associations between demographic characteristics, exposure type, and clinical outcomes were assessed using Pearson’s chi-square test. Statistical significance was evaluated at a two-sided alpha level of 0.05. Pesticide-specific case fatality rates were calculated and used to describe differences in mortality across pesticide categories. To identify factors associated with mortality following pesticide poisoning, a multivariable binary logistic regression model was constructed. Variables included in the model were selected based on epidemiological relevance and availability within the hospital records and comprised sex, age group, exposure type, and pesticide category. Adjusted odds ratios (aORs), 95% confidence intervals, and p-values were reported to quantify associations between predictor variables and mortality outcomes. Prior to model fitting, multicollinearity among predictor variables was assessed using Variance Inflation Factors (VIFs), and no evidence of problematic multicollinearity was observed. Model goodness-of-fit was evaluated using the Hosmer–Lemeshow test, while overall model performance was assessed through examination of residual diagnostics and model calibration. All eligible observations were included in the regression analysis. Missing data were handled using complete-case analysis because the proportion of missing values for variables included in the model was low and there was no evidence of systematic missingness.

### 2.7 Ethical considerations

Ethical approval was obtained from the Kibong’oto–Nelson Mandela–CEDHA Health Research Ethics Committee (KNCHREC) under approval number KNCHREC00034/01/2024. Permission to access hospital records was obtained from participating hospitals and relevant health authorities. Because the study utilized retrospective hospital records, individual informed consent was waived by the ethics committee. All extracted data were anonymized before analysis to protect patient confidentiality.

## 3 Results

### 3.1 Demographic characteristics, exposure patterns, and clinical outcomes

A total of 635 pesticide poisoning cases were included in the analysis. Males accounted for the majority of cases (64.1%; 95% CI: 60.3–67.8), while females represented 35.9% (95% CI: 32.2–39.7). The highest burden of poisoning was observed among individuals aged 16–35 years, who constituted 61.3% of all cases. Within this age range, the 26–35-year age group contributed the largest proportion of cases (31.7%), followed by those aged 16–25 years (29.6%). Intentional poisoning was the predominant mode of exposure, accounting for 446 cases (70.2%; 95% CI: 66.5–73.7), whereas unintentional poisoning accounted for 129 cases (20.3%; 95% CI: 17.3–23.6). Exposure intent could not be determined for 60 cases (9.4%; 95% CI: 7.4–12.0). Intentional poisoning predominated across all adult age groups and was particularly common among individuals aged 16–35 years. In contrast, unintentional poisoning occurred more frequently among younger individuals and constituted a larger proportion of cases among children aged 0–15 years. Males represented the majority of cases in both intentional and unintentional exposure categories. Regarding clinical outcomes, most patients fully recovered following treatment (67.9%; 95% CI: 64.1–71.4). However, 117 patients died, corresponding to an overall case fatality rate of 18.4% (95% CI: 15.5–21.6). Additionally, 7.2% of patients recovered with complications, 3.6% were transferred to higher-level healthcare facilities for further management, and 1.9% absconded before completion of treatment. Chi-square analyses showed no statistically significant associations between sex, age group, or exposure type and clinical outcomes (all p > 0.05), indicating that these demographic and exposure-related characteristics were not significantly associated with differences in treatment outcomes within the study population (Table 1).

**TABLE 1:** Demographic characteristics and associations with clinical outcomes (N = 635).

Characteristic	Category	n (%)	95% CI	p-value
Sex	Male	407 (64.1)	60.3–67.8	0.155
	Female	228 (35.9)	32.2–39.7	​
Age group	0–15	37 (5.8)	4.2–8.0	0.810
	16–25	188 (29.6)	26.2–33.3	​
	26–35	201 (31.7)	28.2–35.4	​
	36–50	153 (24.1)	20.9–27.6	​
	≥50	56 (8.8)	6.9–11.4	​
Exposure type	Intentional	446 (70.2)	66.5–73.7	0.579
	Unintentional	129 (20.3)	17.3–23.6	​
	Unknown	60 (9.4)	7.4–12.0	​
Clinical outcome	Fully recovered	431 (67.9)	64.1–71.4	—
	Recovered with complications	46 (7.2)	5.5–9.5	​
	Death	117 (18.4)	15.5–21.6	​
	Transferred	23 (3.6)	2.4–5.4	​
	Absconded	12 (1.9)	1.1–3.3	​

**FIGURE 1:**
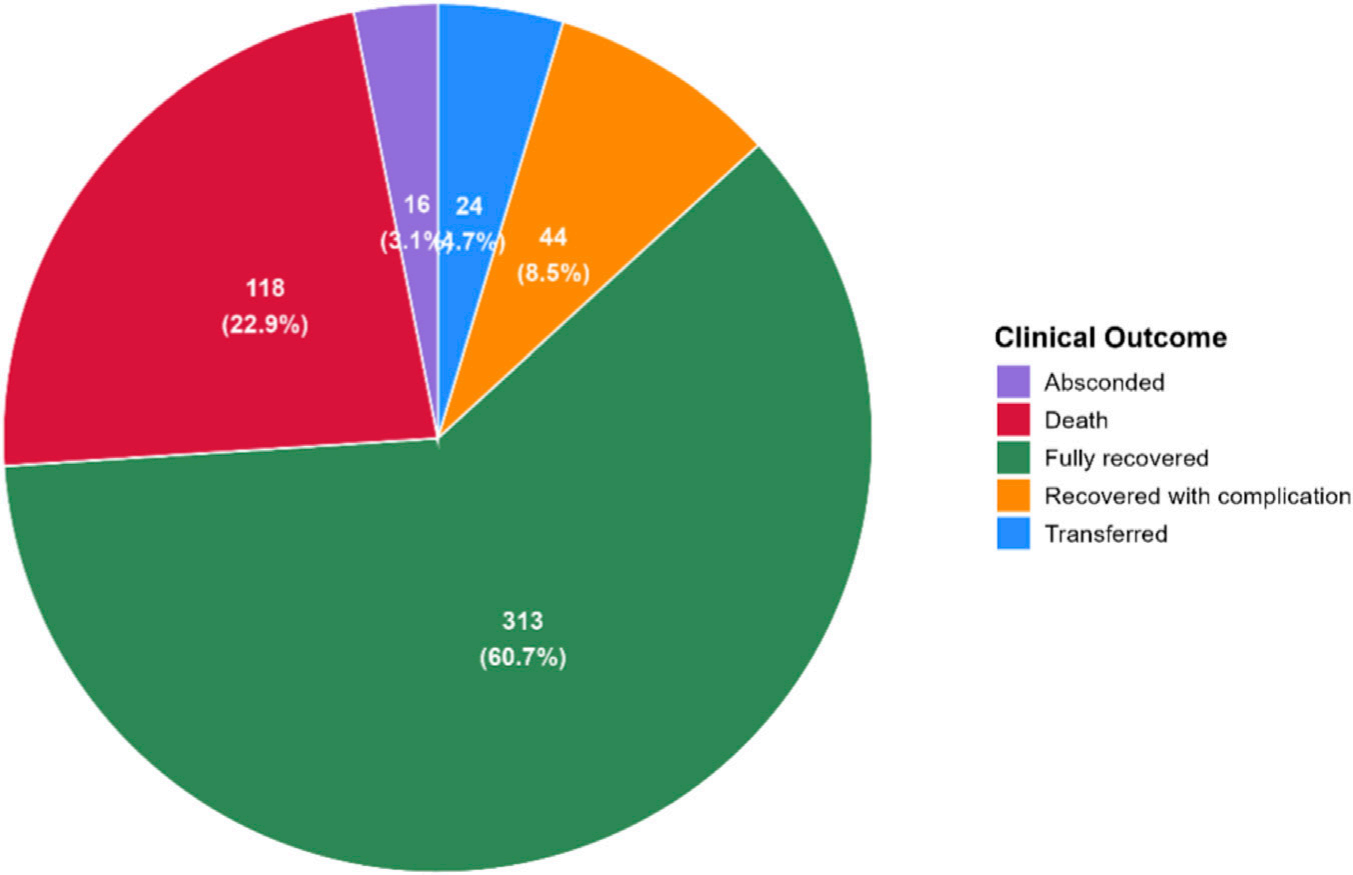
Distribution of clinical outcomes among pesticide poisoning cases.

As illustrated in Figure 1, full recovery was the most common clinical outcome following pesticide poisoning, accounting for more than two-thirds of all cases. Death was the second most frequently reported outcome, highlighting the substantial mortality burden associated with pesticide exposure. Recovery with complications, patient transfers to higher-level facilities, and absconding from treatment were comparatively fewer common outcomes.

### 3.2 Pesticide-specific case fatality and crude mortality risk

**FIGURE 2:**
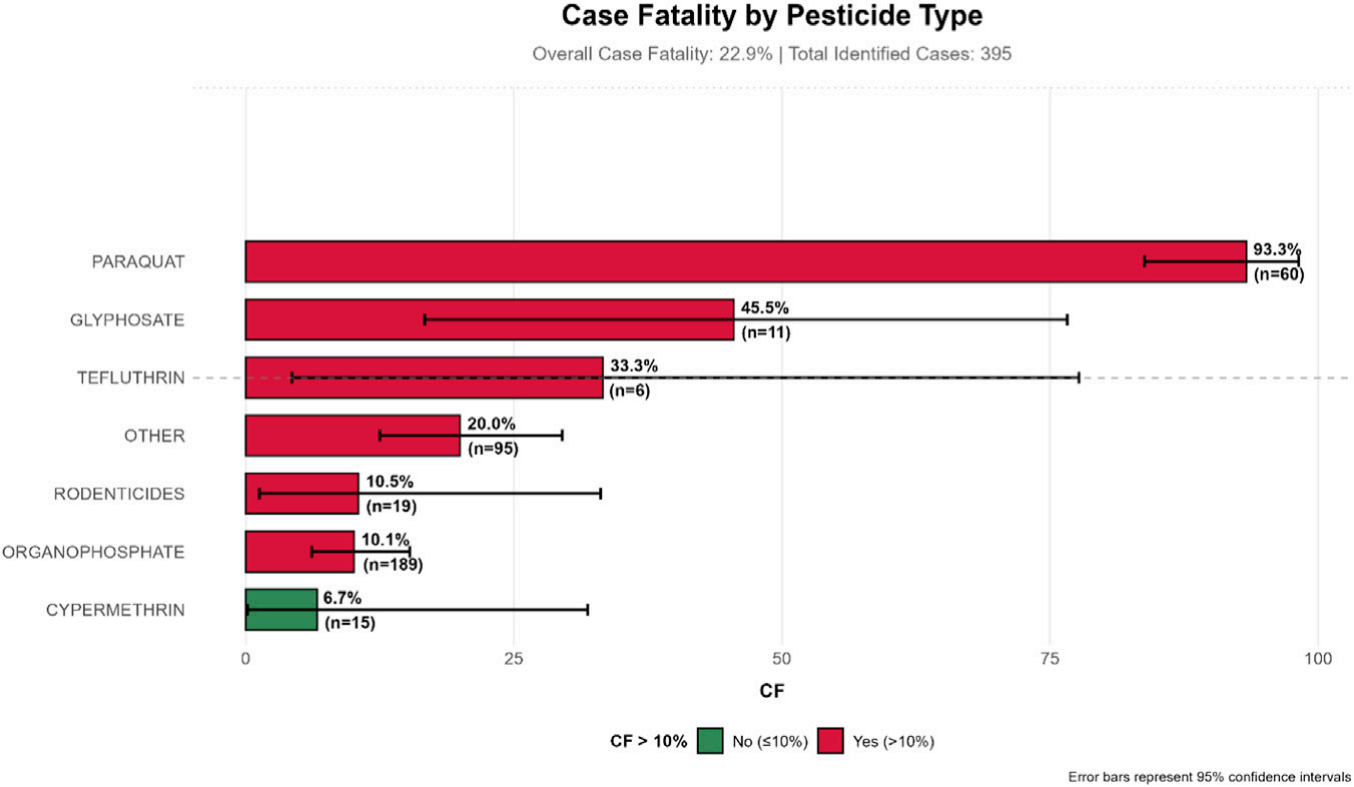
Pesticide-specific case fatality among poisoning cases.

Substantial variation in mortality was observed across pesticide categories (Table 2). Paraquat poisoning demonstrated the highest case fatality rate, with 56 deaths among 60 recorded cases, corresponding to a case fatality of 93.3%. Glyphosate poisoning demonstrated a case fatality of 45.5%, while tefluthrin poisoning showed a case fatality of 33.3%. Organophosphate pesticides represented the largest pesticide category identified in the dataset and accounted for a substantial proportion of poisoning cases. Although their overall case fatality was considerably lower than that observed for paraquat poisoning, organophosphates still contributed substantially to the overall mortality burden. Compared with lower-risk pesticide categories, paraquat poisoning demonstrated markedly elevated crude odds of death (OR = 18.4). Glyphosate poisoning demonstrated a crude odds ratio of 5.8, while tefluthrin poisoning demonstrated a crude odds ratio of 3.2. Because the number of glyphosate (n = 11) and tefluthrin (n = 6) cases was relatively small, the corresponding mortality estimates should be interpreted cautiously. Figure 2 demonstrates substantial variation in case fatality across pesticide categories, with paraquat exhibiting markedly higher mortality than all other pesticide groups.

**TABLE 2:** Pesticide-specific case fatality and crude odds ratios.

Pesticide	Cases	Deaths	Case fatality (%)	Crude OR	Risk category
Paraquat	60	56	93.3	18.4	Extreme
Glyphosate	11	5	45.5	5.8	Extreme
Tefluthrin	6	2	33.3	3.2	High
Organophosphates	189	19	10.1	1.6	High
Rodenticides	19	2	10.5	1.7	High
Cypermethrin	15	1	6.7	References	Moderate

### 3.3 Contribution of individual pesticides to total mortality

**FIGURE 3:**
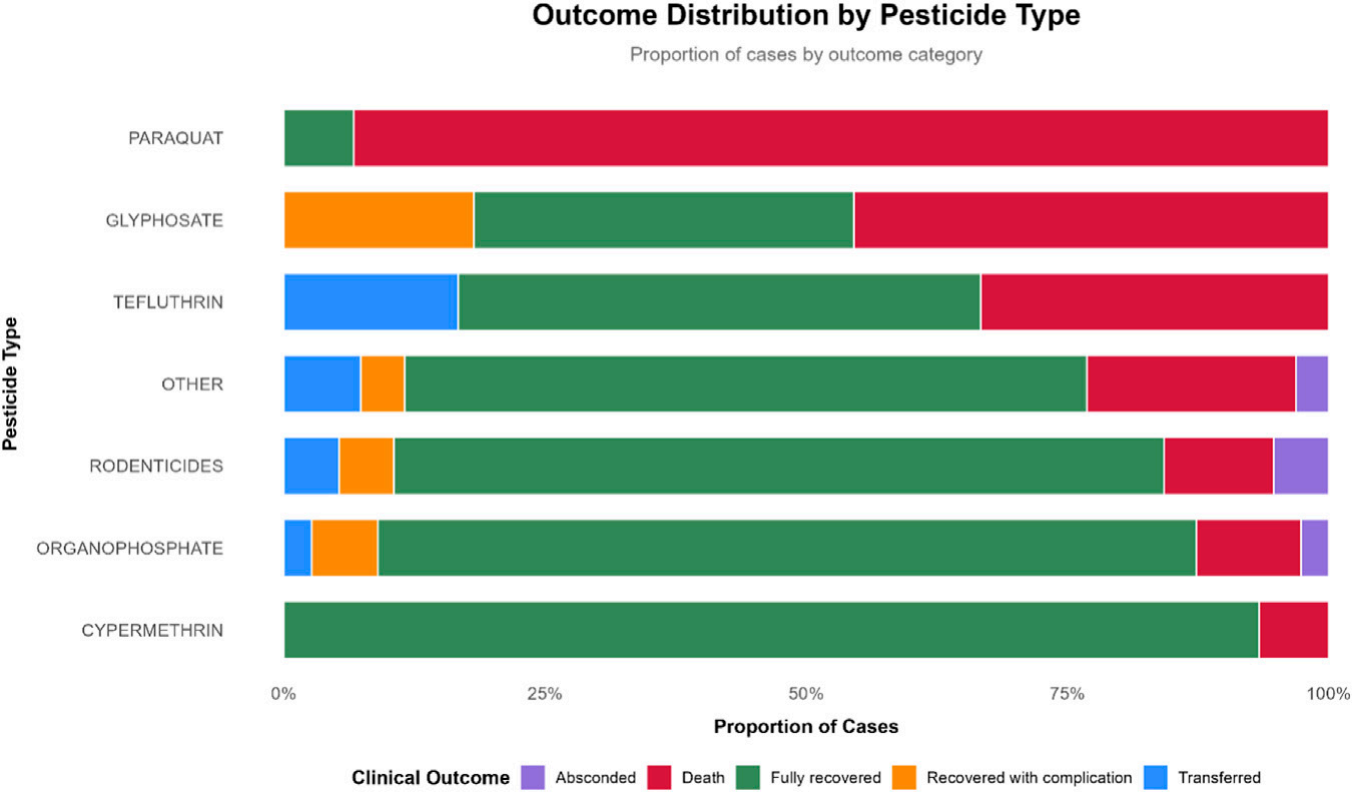
Distribution of pesticide-related deaths by pesticide category.

Paraquat was responsible for nearly half of all pesticide-related deaths recorded during the study period (47.5%; 95% CI: 38.7–56.5), making it the single largest contributor to mortality (Table 3). Organophosphates accounted for 16.1% of all deaths, while other pesticide categories collectively contributed an additional 16.1%. Overall, pesticides with case fatality rates exceeding 10% accounted for 87.3% of all poisoning-related deaths recorded in the study population. Figure 3 illustrates the disproportionate contribution of paraquat to overall mortality compared with all other pesticide categories.

**TABLE 3:** Contribution of pesticide categories to total mortality.

Pesticide category	Deaths	% Of total deaths	95% CI	p-value
Paraquat	56	47.5	38.7–56.5	<0.001
Organophosphates	19	16.1	10.7–23.5	0.041
Other pesticides	19	16.1	10.7–23.5	0.087
Glyphosate	5	4.2	1.8–9.5	0.002

### 3.4 Clinical outcomes among unknown or unspecified pesticides

A total of 102 poisoning cases involved unknown or unspecified pesticide agents. Among these cases, 54.9% fully recovered, while 10.8% resulted in death (Table 4). Recovery with complications and transfer outcomes accounted for 14.7% and 15.7% of cases, respectively. Unknown pesticide classifications may reflect incomplete documentation, inability of severely ill patients to identify the pesticide involved, absence of pesticide containers during hospital presentation, or limitations in pesticide-specific documentation systems.

**TABLE 4:** Clinical outcomes among unknown or unspecified pesticide poisoning cases.

Outcome	n	%	95% CI	p-value
Fully recovered	56	54.9	45.2–64.2	<0.001
Recovered with complications	15	14.7	9.1–22.8	0.214
Death	11	10.8	6.1–18.3	0.087
Transferred	16	15.7	9.9–23.8	0.173
Absconded	4	3.9	1.5–9.7	0.421

### 3.5 Organophosphate poisoning

**FIGURE 4:**
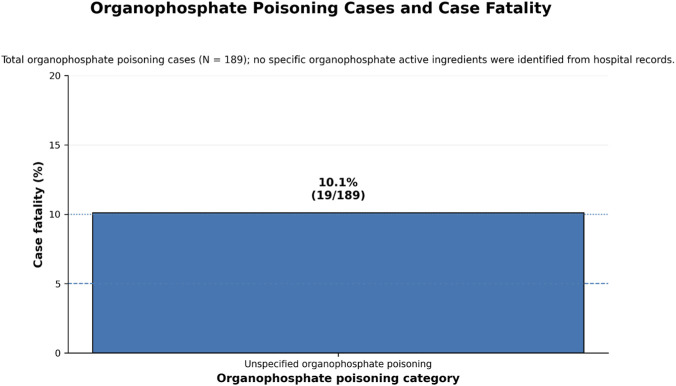
Organophosphate poisoning cases and associated mortality.

A total of 189 organophosphate (OP) poisoning cases were recorded in the medical notes; however, none were identified in hospital records to specific active ingredient level. The overall case fatality among unspecified OP poisoning cases was 10.1% (Table 5). The OP summary presented in Table 5 represents broader unspecified OP records and should not be directly interpreted as identical to the categorized OP cases summarized in Table 2. Figure 4 presents mortality burden among unspecified OP poisoning cases.

**TABLE 5:** Organophosphate poisoning and case fatality.

OP Type	Cases	Deaths	CF (%)
Unspecified OP	189	19	10.1

### 3.6 Predictors of mortality following pesticide poisoning

**FIGURE 5:**
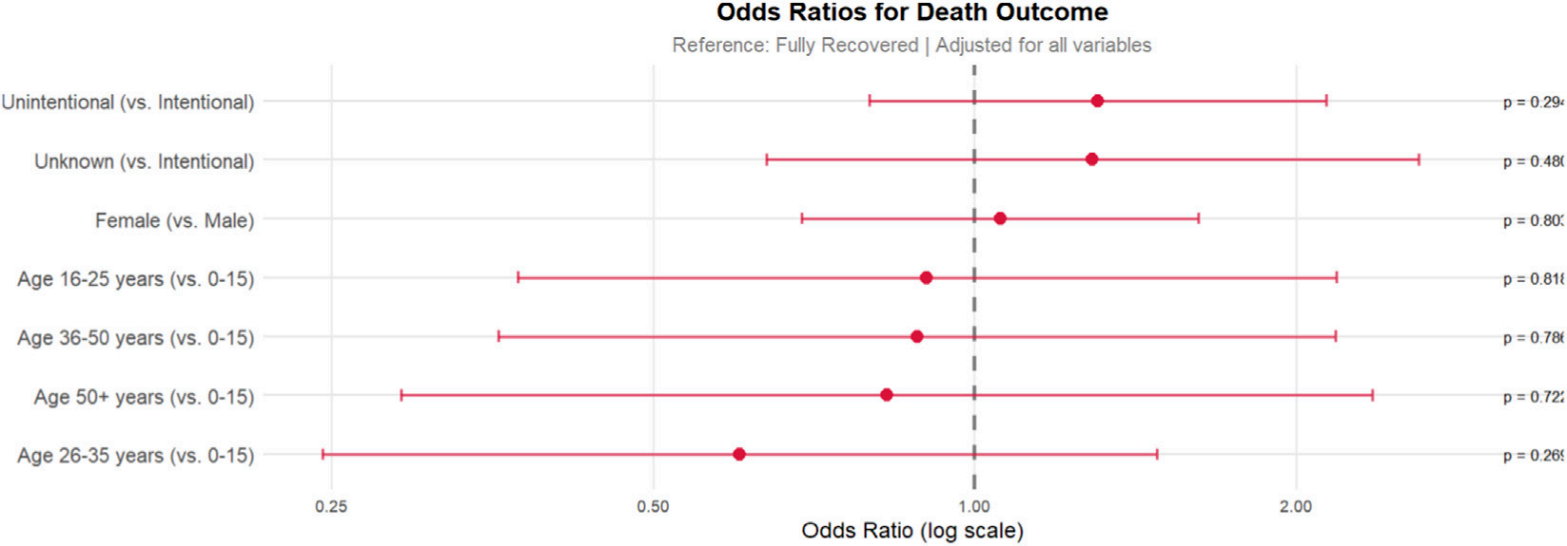
Forest plot showing adjusted odds ratios and 95% confidence intervals for mortality predictors.

Multivariable logistic regression analysis identified substantial differences in mortality risk across pesticide categories (Table 6). No statistically significant associations were observed between mortality and sex, age group, or exposure type. However, pesticide category was strongly associated with mortality. Compared with lower-risk pesticide exposures, paraquat poisoning demonstrated the highest adjusted odds of death (aOR = 21.5; 95% CI: 9.4–48.2; p < 0.001). Glyphosate poisoning was also associated with increased mortality risk (aOR = 5.2; 95% CI: 1.8–14.6; p = 0.002), although this estimate should be interpreted cautiously because of the small number of cases. Organophosphate poisoning demonstrated moderately elevated odds of death (aOR = 1.7; 95% CI: 1.1–2.8; p = 0.041). The forest plot (Figure 5) demonstrates the markedly elevated mortality risk associated with paraquat poisoning compared with other pesticide categories, while demographic characteristics and exposure intent were not significantly associated with mortality.

**TABLE 6:** Multivariable logistic regression analysis of predictors of mortality.

Predictor	Adjusted OR	95% CI	p-value
Female vs. male	1.28	0.87–1.90	0.206
Age 16–25 vs. 0–15	0.73	0.33–1.62	0.441
Age 26–35 vs. 0–15	0.52	0.23–1.15	0.106
Age 36–50 vs. 0–15	0.69	0.31–1.53	0.359
Age ≥50 vs. 0–15	0.71	0.28–1.82	0.479
Unintentional vs. intentional	1.20	0.76–1.92	0.432
Unknown vs. intentional	1.16	0.61–2.22	0.653
Paraquat exposure	21.5	9.4–48.2	<0.001
Glyphosate exposure	5.2	1.8–14.6	0.002
Organophosphate exposure	1.7	1.1–2.8	0.041

## 4 Discussion

This study provides important evidence on pesticide-specific mortality and clinical outcomes among pesticide poisoning cases reported in selected Tanzanian hospitals. The findings demonstrate substantial variation in mortality across pesticide categories and indicate that a relatively small number of highly hazardous pesticides accounted for a disproportionate share of poisoning-related deaths. Most notably, paraquat poisoning exhibited extremely high lethality and was responsible for nearly half of all recorded deaths, highlighting its major contribution to the burden of pesticide poisoning in Tanzania. The observed mortality pattern is consistent with previous international evidence demonstrating that pesticide-specific toxicity is a major determinant of poisoning outcomes (Buckley et al., 2021; Eddleston, 2020). While demographic characteristics and exposure circumstances were not significantly associated with mortality in the present study, substantial differences were observed across pesticide categories. These findings suggest that the intrinsic toxicity of the pesticide involved may be more important in determining survival than demographic factors alone.

Paraquat demonstrated the highest case fatality rate, with 56 deaths among 60 recorded cases. This finding is consistent with numerous studies identifying paraquat as one of the most lethal pesticides following acute poisoning (Buckley et al., 2021; Dinis-Oliveira et al., 2008; Eddleston, 2020). Paraquat poisoning causes severe oxidative damage to multiple organs, particularly the lungs, kidneys, liver, and cardiovascular system (Arsac et al., 2021; Gao et al., 2021; Lovejoy and Fiumera, 2019). Progressive pulmonary fibrosis and multi-organ failure frequently occur despite hospital treatment, contributing to the exceptionally high mortality associated with this compound (Gawarammana and Buckley, 2011). The high case fatality observed in this study may also reflect delayed presentation to health facilities, limited access to advanced supportive care, and the absence of an effective antidote. A particularly important finding was that pesticides with case fatality rates exceeding 10% accounted for approximately 87% of all poisoning-related deaths. This concentration of mortality among a relatively small number of highly hazardous pesticides is consistent with international evidence demonstrating that restriction of specific highly hazardous compounds can substantially reduce poisoning mortality and suicide deaths without adversely affecting agricultural productivity (Laher et al., 2022; Mars et al., 2014). These findings support risk-based pesticide regulation that prioritizes compounds associated with disproportionate human toxicity.

The present findings are particularly relevant in the context of recent regulatory actions undertaken by the Tanzania Plant Health and Pesticides Authority (TPHPA). Tanzania has initiated withdrawal of pesticide products containing active ingredients classified as highly hazardous pesticides, including paraquat-containing formulations and other hazardous compounds. Given that paraquat alone accounted for nearly half of all recorded deaths in this study, continued implementation of these regulatory measures may substantially reduce future pesticide-related mortality. Evidence from several countries has demonstrated significant reductions in pesticide suicide and poisoning mortality following bans or restrictions on highly hazardous pesticides (Laher et al., 2022; Naser Siddique et al., 2024). Continued toxicovigilance and surveillance will be essential to evaluate the public health impact of these interventions in Tanzania. Intentional poisoning accounted for more than two-thirds of all poisoning cases and was particularly common among young adults aged 16–35 years. This finding is consistent with previous studies indicating that pesticides remain a common means of self-harm in agricultural communities because of their widespread availability and high acute toxicity (Mew et al., 2017; World Health Organization, 2021). Young adults represent a socially and economically productive segment of the population; consequently, pesticide-related mortality within this age group may have substantial social and economic consequences. These findings highlight the importance of integrating pesticide regulation with broader mental health interventions, safe storage initiatives, community awareness programmes, and suicide prevention strategies.

Glyphosate poisoning was associated with elevated mortality risk in the regression analysis; however, this finding should be interpreted cautiously. Only 11 glyphosate poisoning cases were identified, resulting in limited statistical precision and potentially unstable effect estimates. Although severe poisoning following ingestion of concentrated glyphosate formulations has been reported in the literature, particularly in intentional self-poisoning cases, the small number of observations in the present study limits definitive conclusions regarding glyphosate lethality in Tanzania. The observed association may reflect severe intentional exposures, formulation-related toxicity, delayed presentation, or random variation arising from the small sample size. Larger studies are required to further evaluate glyphosate-related mortality patterns. Organophosphate pesticides represented the largest pesticide category identified in the study and contributed substantially to the overall burden of poisoning cases. Although mortality associated with organophosphates was lower than that observed for paraquat poisoning, organophosphate exposure remained an important contributor to poisoning-related deaths. Interpretation of these findings is limited because hospital records rarely document specific organophosphate active ingredients. Toxicity varies considerably across organophosphate compounds, and future surveillance systems should prioritize active ingredient-level documentation to facilitate more accurate risk assessment and regulatory decision-making.

This study also identified important gaps in pesticide poisoning surveillance. A considerable proportion of cases involved unknown or unspecified pesticide agents, limiting precise estimation of pesticide-specific mortality patterns. These unknown classifications may reflect incomplete documentation, absence of pesticide containers during presentation, inability of severely ill patients to provide exposure histories, or limitations in hospital recording systems. Similar challenges have been reported in other low-resource settings where toxicology laboratory capacity and poison information services remain limited (World Health Organization, 2020; UNEP, 2022). Strengthening pesticide-specific surveillance and toxicology reporting systems would improve both clinical management and regulatory decision-making. The findings further highlight opportunities for strengthening emergency toxicology services. Although improvements in clinical care may reduce mortality associated with some pesticide poisoning, prevention remains particularly important for highly lethal compounds such as paraquat, where effective treatment options remain limited. Enhancing healthcare worker training, poison information services, referral systems, and clinical toxicology capacity may improve outcomes for pesticide poisoning cases while supporting more effective surveillance and response systems.

Importantly, the observed associations should not be interpreted as evidence of causality. The retrospective observational design permits identification of statistical associations between pesticide categories and mortality outcomes but cannot establish causal relationships. Residual confounding from unmeasured factors may have influenced the observed associations.

### 4.1 Study limitations

#### 4.1.1 Study limitations

Several limitations should be considered when interpreting the findings of this study. First, pesticide identification was based on information documented in hospital records, including patient histories, caregiver reports, referral notes, clinician documentation, and, where available, pesticide containers presented during admission. Because laboratory confirmation of pesticide exposure was not routinely available in participating hospitals, some degree of exposure misclassification may have occurred, potentially affecting pesticide-specific mortality estimates. Second, the retrospective nature of the study limited the availability of important clinical and exposure-related variables that could influence patient outcomes. Information on the quantity of pesticide ingested, route of exposure, time elapsed before hospital presentation, severity of poisoning at admission, treatment administered, access to intensive care support, and duration of hospitalization was not consistently recorded in medical records. The absence of these variables restricted the ability to adjust for all potential confounding factors and may have influenced the observed associations between pesticide categories and mortality.

Third, some pesticide categories were represented by relatively small numbers of cases, particularly glyphosate and tefluthrin poisonings. Consequently, mortality estimates and odds ratios for these pesticide groups should be interpreted with caution because of the limited statistical precision associated with small sample sizes.

In addition, this study relied exclusively on hospital-based data and therefore did not capture poisoning cases or deaths occurring before patients reached healthcare facilities. As a result, the overall burden of pesticide-related mortality may be underestimated. Furthermore, although extensive quality assurance procedures, data cleaning, and record verification were undertaken, retrospective studies are inherently dependent on the completeness and accuracy of existing medical records. Missing information and inconsistencies in documentation may therefore have affected some study findings. Despite these limitations, this study represents one of the largest multi-regional assessments of pesticide-specific poisoning outcomes conducted in Tanzania. The findings provide valuable evidence on pesticide-specific mortality patterns, identify important toxicovigilance gaps, and offer critical information to support pesticide regulation, poisoning prevention strategies, and strengthening of surveillance systems in the country.

## 5 Conclusion

Pesticide poisoning remains an important public health challenge in Tanzania, with mortality strongly concentrated among a small number of highly hazardous pesticides. Paraquat demonstrated exceptionally high case fatality and accounted for nearly half of all recorded pesticide-related deaths, highlighting its disproportionate contribution to poisoning mortality. The findings suggest that substantial reductions in pesticide-related mortality may be achievable through continued restriction and removal of highly hazardous pesticides, particularly compounds associated with disproportionate human lethality. Recent regulatory actions undertaken by the Tanzania Plant Health and Pesticides Authority provide an important opportunity to reduce future poisoning-related morbidity and mortality. Strengthening pesticide-specific surveillance systems, improving toxicovigilance capacity, enhancing healthcare preparedness for poisoning management, and integrating pesticide regulation with mental health and suicide prevention strategies are likely to provide additional public health benefits. Future studies should incorporate prospective data collection, laboratory confirmation of pesticide exposures, and detailed clinical information to better characterize pesticide-specific risks and evaluate the long-term impact of regulatory interventions on poisoning outcomes in Tanzania.

## Data Availability

The raw data supporting the conclusions of this article will be made available by the authors, without undue reservation.
